# Examining Balance and the Likelihood of Falls in Huntington’s Disease

**DOI:** 10.1016/j.prdoa.2025.100399

**Published:** 2025-10-17

**Authors:** Nadeen Youhanan, Japleen Kaur, Andrew Hall, Krisha Bagga, Sean Patel, Anvit Sidhu, Ramez Alskaf, Zafeer Shaik, Paul E. Gilbert, Daniel J Goble, Jody Corey-Bloom

**Affiliations:** a Neurosciences, UC San Diego, CA, the United States of America; b Department of Psychology, San Diego State University, CA, the United States of America; c Exercise Science, Oakland University, Rochester MI, the United States of America

## Abstract

**Background:**

Balance impairment may begin prior to motor diagnosis in Huntington’s disease (HD) and is associated with an increased risk of falls—an important predictor of nursing home placement.

**Objective:**

To evaluate the ability of three balance measures to discriminate between Fallers and Non-Fallers and to estimate the likelihood of falling in HD patients.

**Methods:**

125 gene-positive individuals were stratified into Fallers (n = 30) and Non-Fallers (n = 95) based on fall history. Participants completed a Total Body Sway (TBS) assessment using the BTrackS™ Balance Plate, Timed Up-and-Go (TUG), and Chair Sit-to-Stand Test (CST). Group differences were analyzed with Mann–Whitney *U* test. ROC curve analysis was used to calculate likelihood ratios (LRs) for each assessment’s ability to distinguish Fallers from Non-Fallers.

**Results:**

Fallers demonstrated significantly higher TBS scores compared to Non-Fallers (p < 0.001), while differences on TUG (p = 0.098) and CST (p = 1.00) were not significant. TBS yielded the highest area under the curve (AUC = 0.89, p < 0.001), followed by TUG (AUC = 0.66, p = 0.008), while CST did not discriminate between groups (AUC = 0.47, p = 0.617). TBS also demonstrated superior diagnostic utility (LR+ = 5.06; LR– = 0.24) compared to TUG (LR+ = 3.41; LR– = 0.62) and CST (LR+ = 1.59; LR– = 0.90).

**Conclusion:**

TBS and, to a lesser extent, TUG are valid tools for identifying individuals at risk of falling in HD. In contrast, the CST may not be an effective clinical measure in this regard.

## 1 Introduction

Huntington’s Disease (HD) is an autosomal-dominant neurodegenerative disease characterized by expanded trinucleotide CAG repeats in the *Huntingtin* (HTT) gene on chromosome 4 [1]. CAG repeat lengths of 39 or greater lead to increased mutant Huntingtin (mHTT) protein production [2]. The mHTT protein affects neuronal function, most notably in the basal ganglia, cerebral cortex, and striatum. Dysfunction in the basal ganglia, the brain region responsible for motor control and balance, leads to pronounced motor symptoms, including bradykinesia, chorea, and balance impairment. Currently, in addition to gene-positive status and family history of HD, clinical diagnosis is largely based on the onset of motor symptoms [1].

Balance impairment, defined as the reduced ability to maintain an upright stance [3], is a key feature of motor dysfunction in HD. A recent study from our laboratory examining balance in HD identified notable differences between manifest HD patients and normal controls [4]. Further, additional studies have shown that balance impairment may begin in the premanifest stage and worsen as patients transition to manifest HD [[4], [5], [6]].

A variety of balance assessments have been used to investigate balance dysfunction in HD. One study examined the sensitivity of the Berg Balance Scale (BBS), Functional Reach Test (FRT), and Timed Up-and-Go (TUG) to disease progression in HD [7]. The BBS, FRT, and TUG assessments were all able to differentiate between three levels of functioning on the Total Functional Capacity Scale (TFC). Another study utilizing the Mini-BESTest found that individuals with HD exhibited significantly greater balance impairment than healthy controls [8].

Alternatively, Total Body Sway (TBS)—a quantitative measure of postural stability obtained from the BTrackS™ Balance System, which assesses the overall displacement (in centimeters) of a person’s center of pressure—has also been used to objectively evaluate balance. While a previous study from our laboratory measured TBS with a Wii Balance Board [4], this device was not designed for clinical use. In this case, the BTrackS^TM^ Balance Plate [3,9] is considered a more suitable tool for assessing TBS. Indeed, our laboratory has previously used the BTrackS^TM^ Balance Plate to examine balance impairment within a cognitive-motor dual task paradigm [10].

Balance impairment has been linked to an increased risk of falls in HD by a number of studies utilizing the TUG, BBS, and Tinetti Mobility Test (TMT) to measure the association between falls and balance impairment [7,11,12]. Investigating the likelihood of falls in HD is important as a history of falls is the primary predictor of nursing home placement [13]. Since previous literature has shown an association between balance impairment and a history of falls, we reasoned that balance impairment might aid in predicting likelihood of falls [7,11,12]. Therefore, the purpose of our current study was to examine balance in HD using several established balance measures in a well-characterized cohort of gene-positive individuals, identified as Fallers or Non-Fallers, on the basis of fall history.

## 2 Methodology

This is a cross-sectional observational study. We recruited 130 gene-positive subjects at the University of California, San Diego Huntington’s Disease Clinical Research Center (HDCRC) and HDSA Center of Excellence. Of these, 63 were manifest HD, a motor diagnosis defined as a diagnostic confidence level (DCL) of 4 (unequivocal motor signs, ≥99 % confidence) on the standardized motor exam of the Unified Huntington Disease Rating Scale (UHDRS) and 67 were pre-manifest (PM) with a DCL < 4. A ROUT outlier analysis, in addition to neurologist input, excluded 1 HD and 4 PM patients.

Participants were stratified on the basis of history of falls. Subjects who had not fallen within the past 12 months were classified as Non-Fallers (n = 95), while subjects who had fallen at least once during the 12 months prior to visit were classified as Fallers (n = 30). Exclusion criteria included neuropathy and age > 64 years due to potential age-related balance issues unrelated to HD. All participants were provided written and informed consent in accordance with the Institutional Review Board (IRB) Committee (IRB Protocol #170038).

### 2.1 Assessments

Subjects were administered the UHDRS, including TFC, Total Motor Score (TMS), and Total Maximal Chorea (TMC) Score. In addition, they were administered the Symbol Digit Modality Test (SDMT), Montreal Cognitive Assessment (MoCA), and the Mini-Mental State Examination (MMSE). The composite UHDRS (cUHDRS) score was calculated for each participant. Balance assessments included the TUG, Chair Sit-to-Stand (CST), and the BTrackS™ Balance Test. Since it has been suggested that Body Mass Index (BMI) may have an influence on center of pressure [14], BMI was also calculated for all participants. To determine fall history, subjects and their caregiver were asked at the time of visit whether the patient had fallen within the past year.

### 2.2 BTrackS™ Balance Plate

The BTrackS™ Balance Plate is a FDA registered, lightweight (<7 Kg) force plate specialized for determining the center of pressure (COP) of foot forces placed on it during standing. The surface of the BTrackS™ Balance Plate measures 0.4  m by 0.6  m and uses four-sensor technology to determine COP. The BTrackS™ Balance Plate used in this study was placed on a firm, level surface during testing, as per the manufacturer's specifications. Leveling of the board was achieved via the adjustable legs on the BTrackS™ Balance Plate and verified with a bubble leveling tool. The BTrackS™ Sport Balance software is an application-based program that has been loaded onto an ASIS laptop (Model X200) with a Windows 8.1 operating system and is connected to the laptop via a USB cable.

The BTrackS™ Balance Plate was used to measure Total Body Sway (TBS) for each subject as the total centimeter deviation from their starting COP. Subjects removed their shoes prior to standing on the device. They stood on the balance plate with their eyes open, hands on their hips, feet shoulder width apart, and head straight. The average TBS of four ten second trials was calculated.

### 2.3 Likelihood Ratio

To assess the diagnostic accuracy of balance measures in identifying individuals at risk of falling, likelihood ratios (LRs) were calculated [15]. LRs have been widely used in previous studies to evaluate the clinical utility and diagnostic performance of assessment tools [12,16]. LRs are derived from a test’s sensitivity and specificity. Sensitivity refers to the test’s ability to correctly identify true positives (Fallers), while specificity refers to its ability to correctly identify true negatives (Non-Fallers).

Two types of LRs are calculated: the positive likelihood ratio (LR + ) and the negative likelihood ratio (LR–). LR + indicates how much more likely a positive test result is to occur in someone who is a Faller compared to someone who is not. LR– reflects how much more likely a negative test result is to occur in someone who is not a Faller compared to someone who is. The formulas used are:

• LR+ = Sensitivity / (1 – Specificity)
• LR– = (1 – Sensitivity) / Specificity

In our analysis, an LR + greater than 1 and/or an LR– less than 1 indicates that the measure has some ability to distinguish Fallers from Non-Fallers. We interpreted likelihood ratios using the following guidelines for clinical utility [16]:

• LR+ >10 **=** strong evidence to rule in Fallers
• LR + 5–10 = moderate evidence
• LR + 2–5 = small or weak evidence
• LR + 1–2 = minimal to no added value
• LR– <0.1 = strong evidence to rule out Fallers
• LR– 0.1–0.2 = moderate evidence
• LR– 0.2–0.5 = small or weak evidence
• LR– 0.5–1.0 = minimal to no added value

### 2.4 Statistical Analysis

Data was analyzed using GraphPad Prism 9.5.1 for Macintosh (GraphPad Software, La Jolla, CA, USA) and SPSS Statistics for Macintosh (IBM Corp. Armonk, NY, USA). A Shapiro-Wilk test indicated that the data were not normally distributed (p < 0.01), therefore, a non-parametric Mann-Whitney *U* test with Bonferroni correction was used to compare Fallers and Non-Fallers. Effect sizes were calculated using Cohen’s d (d). Nonparametric Receiver Operating Curve (ROC) analysis with Youden’s Index was conducted to assess the discriminatory power of multiple balance measures and to calculate the likelihood ratio (LR) for distinguishing between Fallers and Non-Fallers.

## 3 Results

### 3.1 Clinical and Demographic Characteristics

One PM and 29 manifest HD subjects comprised the Fallers group, while 62 PM and 33 manifest HD subjects comprised the Non-Fallers group. Clinical and demographic characteristics are summarized in Table 1. Fallers and Non-Fallers were well-matched for age (p = 0.195), gender (p = 1.00), education (p = 1.00), and BMI (p-value = 1.00). In this case, these variables were not included as covariates in subsequent analyses.

**Table 1:** Clinical and Demographic Characteristics of Fallers and Non-Fallers. Mean (SD). Cohort performance compared using nonparametric Independent Mann-Whitney U Test with Bonferroni Correction. Effect Size measured using Cohen’s D.

	**Fallers**	**Non-Fallers**	p-value*	**Effect Size (d)**
**n**	30	95	−	−
**Age [years]**	49.03 (8.68)	43.33 (12.17)	0.210	−
**Gender [M/F]**	12/18	49/46	1.00	−
**Education [years]**	15.07 (2.84)	15.43 (2.63)	1.00	−
**BMI**	24.04 (4.11)	25.83 (4.65)	1.00	−
**MoCA**	22.23 (6.03)	26.24 (3.30)	<0.01	0.98
**MMSE**	25.10 (5.34)	27.45 (2.23)	0.35	0.73
**SDMT**	30.17 (14.14)	45.47 (13.74)	<0.001	1.11
**TFC**	9.13 (2.92)	12.23 (1.40)	<0.001	1.65
**TMS**	23.93 (14.89)	8.98 (10.83)	<0.001	1.26
**TMC**	5.03 (3.27)	1.79 (2.50)	<0.001	1.20
**TUG [s]**	12.30 (5.12)	9.87 (2.09)	0.098	0.79
**CST**	12.73 (4.66)	12.47 (4.02)	1.00	0.06
**TBS [cm]**	26.49 (19.61)	11.05 (4.31)	<0.001	1.51
**cUHDRS**	11.55 (3.65)	15.67 (2.69)	<0.001	1.40

BMI = Body Mass Index; MoCA = Montreal Cognitive Assessment; MMSE = Mini Mental State Examination; SDMT = Symbol Digit Modality Test; TFC = Total Functional Capacity; SWR = Stroop Word Reading; TMS = Total Motor Score; TMC = Total Maximal Chorea; TUG = Timed Up-and-Go; CST= Chair Sit-to-Stand; TBS = Total Body Sway; cUHDRS = composite Unified Huntington’s Disease Rating Scale. *Adjusted p-value using Bonferroni.

### 3.2 Characteristics of Fallers

**Fig. 1:**
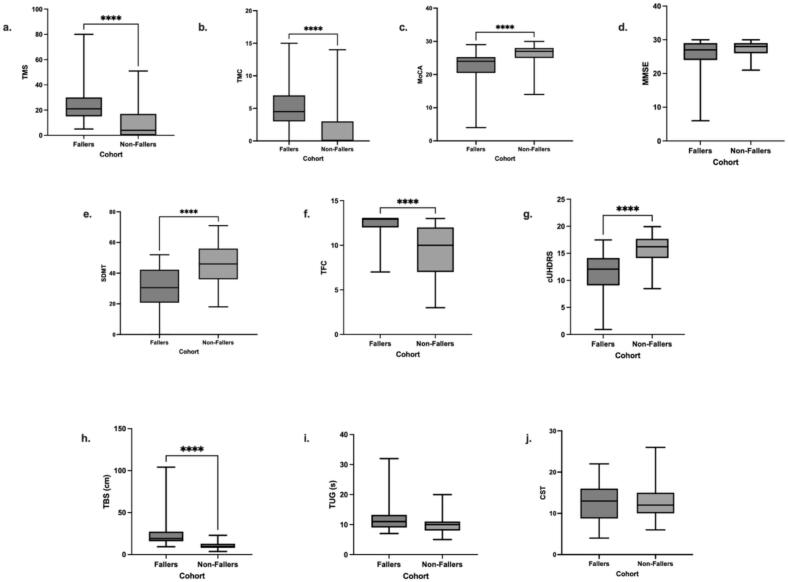
Nonparametric Independent Mann Whitney-U T-Test adjusted with Bonferroni correction between Fallers and Non-Fallers. (**a**) Total Motor Score (TMS); (**b**) Total Maximal Chorea (TMC); (**c**) Montreal Cognitive Assessment (MoCA); (**d**) Mini Mental State Examination (MMSE); (**e**) Symbol Digit Modality Test (SDMT), (**f**) Total Functional Capacity (TFC); (**g**) composite Unified Huntington’s Disease Rating Scale (cUHDRS); and (**h**) Total Body Sway (TBS); (**i**) Timed Up-and-Go (TUG); (**j**) 30-Second Chair Sit-to-Stand (CST).

Motor function was examined using TMS and TMC scores. Fallers exhibited greater motor impairment- more than double the TMS (23.93 vs 8.98, respectively; p < 0.001, d = 1.26) and more than double the TMC (5.03 vs 1.79 respectively; p < 0.001, d = 1.20) of Non-Fallers (Fig. 1a-b).

Cognitive ability was assessed using the Montreal Cognitive Assessment (MoCA), the Symbol Digit Modalities Test (SDMT), and the Mini-Mental State Examination (MMSE). Fallers performed significantly worse than Non-Fallers on the MoCA (p < 0.01, d = 0.98) and SDMT (p < 0.001, d = 1.11), but not on the MMSE (p = 0.35, d = 0.73) (Fig. 1c-e).

Functional loss was assessed using the TFC. Fallers demonstrated significantly greater impairment compared to Non-Fallers (9.13 vs. 12.23; p < 0.0001, d = 1.65) (Fig. 1f).

The cUHDRS scores, a composite measure of motoric, cognitive, and functional abilities, were significantly lower (worse) in Fallers than in Non-Fallers (p < 0.001, d = 1.40) (Fig. 1g).

### 3.3 Comparing Balance Measures

Balance proficiency was assessed using the TBS, TUG, and CST. Fallers demonstrated significantly greater balance impairment on the TBS (p < 0.001), with a large effect size (d = 1.51) compared to Non-Fallers, indicating that the TBS effectively differentiated between the two groups (Fig. 1h). In contrast, neither the TUG (p = 0.098, d = 0.79) nor the CST (p = 1.00, d = 0.06) showed statistically significant differences between Fallers and Non-Fallers, and both exhibited small effect sizes (Fig. 1i-j). These results support the superior discriminative ability of the TBS in identifying individuals at risk of falling.

### 3.4 Likelihood Ratios between Fallers and Non-Fallers

**Fig. 2:**
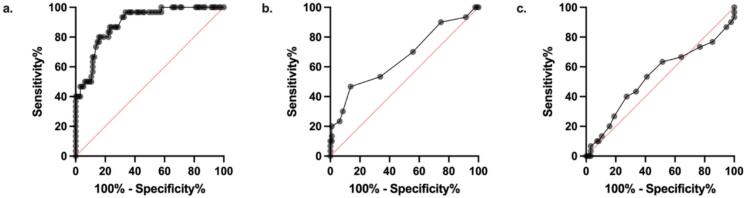
Nonparametric Receiver Operator Characteristic (ROC) curve between Fallers and Non-Fallers. (**a**) Total Body Sway (TBS), p-value < 0.0001, Area Under the Curve (AUC) = 0.89, Positive Likelihood Ratio (LR + ) = 5.06, Negative LR (LR-) = 0.24; (**b**) Timed Up-and-Go (TUG), p-value = 0.008, AUC = 0.66, LR+ = 3.41, LR- = 0.62; and (**c**) 30 Second Chair Sit-to-Stand (CST), p-value = 0.617, AUC = 0.47, LR+ = 1.59, LR- = 0.90.

ROC analyses (Fig. 2) were conducted to evaluate the ability of the three balance measures −TBS, TUG, and CST −to discriminate between Fallers and Non-Fallers and to estimate the LR associated with fall risk. TBS demonstrated the strongest discriminatory power, with an area under the curve (AUC) of 0.89 (p < 0.001) and an optimal cut-off score of 15.25 cm, indicating excellent accuracy in identifying individuals at risk of falling. The TUG showed moderate discriminatory ability, with an AUC of 0.66 (p = 0.008) and a cut-off value of 11.50 s. In contrast, the CST did not significantly differentiate Fallers from Non-Fallers, yielding a non-significant AUC of 0.47 (p = 0.617) and a cut-off value of 8.50.

Using ROC-derived Youden’s Index, optimal thresholds were identified for each balance measure to maximize combined sensitivity and specificity. These values were then used to calculate corresponding likelihood ratios (Table 2). TBS demonstrated strong diagnostic performance, with a sensitivity of 80.0 % and specificity of 84.2 %, resulting in a positive likelihood ratio (LR^+^) of 5.06 and a negative likelihood ratio (LR^–^) of 0.24. The TUG showed lower sensitivity (46.7 %) but retained high specificity (86.3 %), yielding an LR^+^ of 3.41 and an LR^–^ of 0.62. In contrast, the CST demonstrated limited diagnostic value, with low sensitivity (23.3 %) despite high specificity (85.3 %), resulting in an LR^+^ of 1.59 and an LR^–^ of 0.90.

**Table 2:** Discriminative Validity of Balance Assessments Based on ROC Analysis.

**Assessment**	**AUC**	**p-value**	**Sensitivity (%)**	**Specificity (%)**	**LR+**	**LR-**	**Cut-off Value**
**TBS**	0.89	<0.001	80.00	84.20	5.06	0.24	15.25
**TUG**	0.66	0.008	46.70	86.30	3.41	0.62	11.50
**CST**	0.47	0.617	23.30	85.30	1.59	0.90	8.50

AUC = Area Under the Curve; TBS = Total Body Sway; TUG = Timed Up-and-Go; CST = Chair Sit-to-Stand.

## 4 Discussion

The objectives of this study were to examine the characteristics of HD gene positive individuals who fall, compare the discriminative ability of several common balance measures in identifying Fallers vs. Non-Fallers, and determine which of the three tools most effectively identifies individuals at risk of falling. We found that gene positive individuals who fall exhibit more motor, cognitive, and functional impairment than those who do not. In addition, TBS demonstrated superior discriminative ability in identifying individuals at risk of falling. Finally, based on likelihood ratios derived from ROC analyses, both the TBS and, to a lesser extent, the TUG identified individuals at increased fall risk.

Our findings regarding the clinical characteristics of Fallers versus Non-Fallers are consistent with prior literature, despite the use of different balance assessments. Grimbergen et al. (2008), using the BBS, found that increased motor impairment, poor postural control, and lower SDMT and TFC scores were associated with a greater likelihood of falling in individuals with HD—a pattern echoed in our data [17]. Similarly, Fritz et al. (2016) reported that executive dysfunction and attentional deficits were common among HD Fallers, with cognitive performance on the SDMT and MoCA showing strong correlations with fall risk [18].

In our ROC analysis, both the TBS and the TUG test were able to discriminate between Fallers and Non-Fallers, with the TBS showing superior discriminatory power (AUC = 0.89) compared to the TUG (AUC = 0.66). In contrast, the CST did not differentiate between the groups (AUC = 0.47, not significant). These findings align with those of Busse et al. (2009), who evaluated the performance of the TUG and BBS in a small HD cohort (n = 24), including 14 Recurrent Fallers and 10 Non-Fallers. Their results demonstrated that both balance measures effectively distinguished between Fallers and Non-Fallers.

The likelihood ratios derived from the ROC analyses provide insight into the clinical utility of each balance measure for identifying individuals at increased risk of falling. The TBS, with an LR^+^ of 5.06, offers moderate diagnostic evidence to rule in fall risk, meaning that individuals who score below the TBS threshold are over five times more likely to be Fallers than Non-Fallers. In contrast, the TUG, while highly specific, demonstrated only weak diagnostic utility overall (LR^+^ = 3.41; LR^–^ = 0.62), limiting its ability to confidently identify or exclude individuals at risk. The CST, with an LR^+^ of 1.59 and LR^–^ of 0.90, offers minimal discriminatory value, suggesting that it may not be a reliable measure for fall risk screening in this population.

Taken together, the current findings highlight TBS as the most effective of the three evaluated tools for identifying individuals at risk of falling. Its high sensitivity and specificity, coupled with a positive likelihood ratio exceeding 5, indicate that it can reliably distinguish Fallers from Non-Fallers. The moderate diagnostic strength of the TBS supports its role not only as a screening tool, but also as a guide for targeting individuals for early intervention and fall prevention efforts. In contrast, while the TUG test demonstrated acceptable specificity, its limited sensitivity and weaker likelihood ratios diminish its utility in confidently identifying high-risk individuals. The CST, offering minimal diagnostic value, appears unsuitable as a stand-alone measure in this context. Incorporating the TBS into routine clinical evaluations may enhance early detection and intervention strategies, ultimately reducing fall-related morbidity in at-risk HD populations. In the future, new technologies, such as PC-based methods and transcranial magnetic stimulation, may be utilized to decrease fall risk in neurological patients [19].

Nevertheless, this study is not without limitations. First, we did not perform a prospective sample size calculation; rather, analyzed a consecutive convenience sample of all gene-positive HD participants who met eligibility criteria during the study period. Second, the Fallers cohort was modest; however, our large observed effect sizes and narrow confidence intervals across measures support the robustness of our conclusions. Additionally, we did not include other widely used balance measures, such as the BBS or the TMT, which limits our ability to compare their clinical utility in predicting fall risk in HD. Finally, although the TBS demonstrated the strongest discriminatory ability in our HD sample, the BTrackS™ Balance Plate—while readily available—is not widely used in many clinical settings. Future studies should prioritize evaluating objective balance measures that are more feasible for use in typical clinical and outpatient environments.

## 5 Conclusion

The presence of balance impairment may aid in predicting the likelihood of falls in HD. Use of objective balance measures, particularly the TBS, followed by the TUG, but not the CST, appear to better differentiate between Fallers and Non-Fallers in individuals positive for the HD gene.

## CRediT authorship contribution statement

**Nadeen Youhanan:** Writing – review & editing, Writing – original draft, Visualization, Validation, Software, Methodology, Formal analysis, Data curation, Conceptualization. **Japleen Kaur:** Writing – review & editing, Writing – original draft, Supervision, Methodology, Investigation, Formal analysis, Conceptualization. **Andrew Hall:** Writing – original draft, Methodology, Investigation, Data curation, Conceptualization. **Krisha Bagga:** Writing – review & editing, Writing – original draft, Validation, Supervision, Project administration, Methodology. **Sean Patel:** Supervision, Investigation, Data curation. **Anvit Sidhu:** Methodology, Investigation, Data curation. **Ramez Alskaf:** Methodology, Investigation, Data curation. **Zafeer Shaik:** Investigation, Data curation. **Paul E. Gilbert:** Writing – review & editing, Visualization, Conceptualization. **Daniel J Goble:** Writing – review & editing, Supervision. **Jody Corey-Bloom:** Writing – review & editing, Writing – original draft, Validation, Supervision, Software, Resources, Project administration, Methodology, Investigation, Formal analysis, Conceptualization.

## Declaration of competing interest

DJG is eligible for royalties from a patent (OMB 0651-0032) related to the technology used in this study. In addition, he has an equity stake (stock options) in Balance Tracking Systems, Inc. This financial conflict of interest is mitigated by a management plan put in place by his academic institution to ensure the integrity of his research. The authors declare no other conflicts of interest in this work.
