# Corrigendum to “Examining balance and the likelihood of falls in Huntington’s disease”. [Clin. Parkinsonism Relat. Dis. 13 (2025) 100399]

**DOI:** 10.1016/j.prdoa.2025.100408

**Published:** 2025-11-22

**Authors:** Nadeen Youhanan, Japleen Kaur, Andrew Hall, Krisha Bagga, Sean Patel, Anvit Sidhu, Ramez Alskaf, Zafeer Shaik, Paul E. Gilbert, Daniel J. Goble, Jody Corey-Bloom

**Affiliations:** a Neurosciences, UC San Diego, CA, USA; b Department of Psychology, San Diego State University, CA, United States; c Exercise Science, Oakland University, Rochester MI, USA

The authors regret an error in Figure 1 of the original publication. Figure 1f cohorts were mislabelled in the original submission. We have rectified this error and submit the corrected Figure 1 below.

The authors would like to apologise for any inconvenience caused.

**Figure:**
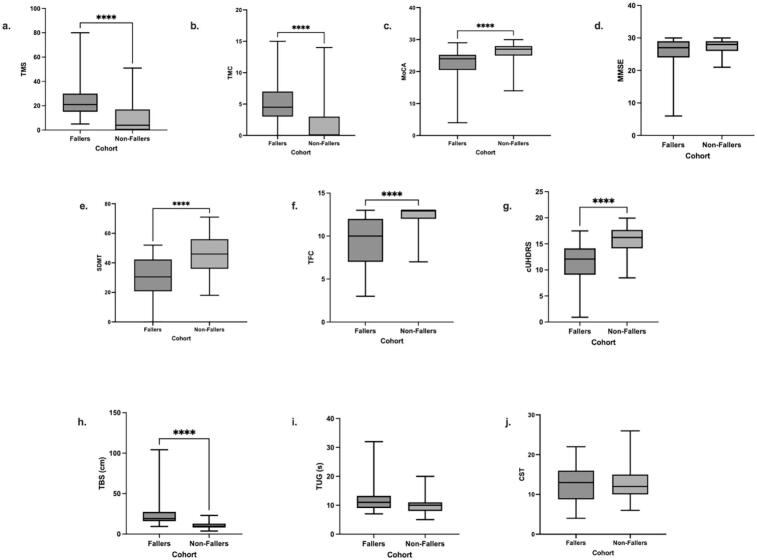

**Figure 1.** Nonparametric Independent Mann Whitney-U T-Test adjusted with Bonferroni correction between Fallers and Non-Fallers. (**a**) Total Motor Score (TMS); (**b**) Total Maximal Chorea (TMC); (**c**) Montreal Cognitive Assessment (MoCA); (**d**) Mini Mental State Examination (MMSE); (**e**) Symbol Digit Modality Test (SDMT), (**f**) Total Functional Capacity (TFC); (**g**) composite Unified Huntington’s Disease Rating Scale (cUHDRS); and (**h**) Total Body Sway (TBS); (**i**) Timed Up-and-Go (TUG); (**j**) 30-Second Chair Sit-to-Stand (CST).

